# Bedside Small-Bowel Challenge vs. Fluoroscopic Series for SBO: A Cost Effectiveness Analysis

**DOI:** 10.3390/tomography11100107

**Published:** 2025-09-26

**Authors:** Aravinda Krishna Ganapathy, Liam Cunningham, M. Hunter Lanier, Selasi Nakhaima, Madelyn Thiel, Daniel Hoffman, Obeid Ilahi, David H. Ballard, Vincent M. Mellnick

**Affiliations:** 1 School of Medicine, Washington University in St. Louis, St. Louis, MO 63110, USA; 2 Mallinckrodt Institute of Radiology, Washington University School of Medicine, St. Louis, MO 63110, USA; 3 Barnes Jewish Hospital, St. Louis, MO 63110, USA; 4 Department of Surgery, Washington University School of Medicine, St. Louis, MO 63110, USA

**Keywords:** small bowel obstruction, health care costs, resource utilization, X-ray, bedside imaging, fluoroscopy, abdominal radiology

## Abstract

Background: Small bowel obstruction (SBO) accounts for 12–16% of surgical hospital admissions and can lead to complications such as bowel ischemia. Traditional management requires transporting patients to the Radiology Department (RD) for a fluoroscopic small bowel series, occupying resources and time. This study evaluates the efficacy and efficiency of the Small Bowel Challenge Exam, a bedside alternative. Methods: A retrospective analysis was performed on 85 SBO patients from January 2018 to December 2023 at an academic tertiary care facility, comparing the traditional fluoroscopic series (37 patients) to the bedside Small Bowel Challenge Exam (48 patients). Key metrics analyzed included hospital resource utilization, overall costs, and length of stay. Results: Gender and race distributions were similar between groups (*p* = 0.268 and *p* = 0.808, respectively). Median total costs were lower in the challenge group (USD 1243 vs. USD 1472; *p* = 0.1229), significantly so when excluding CT scan costs (USD 993.5 vs. USD 1270; *p* = 0.0500). Core costs also significantly favored the challenge group (USD 389.6 vs. USD 615; *p* < 0.0001). Length of stay and variable costs showed no significant differences (*p* = 0.3846 and *p* = 0.8065, respectively). Additional imaging frequencies were comparable (*p* = 0.96 for CT scans; *p* = 0.97 for XR exams). Conclusions: The Small Bowel Challenge Exam reduces certain costs and logistical burdens without prolonging length of stay, suggesting more efficient use of hospital resources. Further research is recommended to evaluate broader implementation and long-term impacts.

## 1. Introduction

Small bowel obstruction (SBO) occurs when there is a mechanical blockage in the small intestine, often presenting as an acute abdomen. SBO has continued to be a major cause of hospitalizations, contributing to 12–16% of surgical admissions [1] with acute abdominal conditions in the United States. Cases most often occur in the setting of postoperative abdominal adhesions, contributing to up to 75% of cases in the industrialized world, but are also known to be the result of hernias or malignancies [2,3]. To our knowledge, no prior study has juxtaposed bedside challenge and fluoroscopic series using real-world cost accounting and resource utilization metrics at a tertiary center.

Prompt diagnosis and timely management of SBO are critical in reducing morbidity and mortality. Delayed or missed recognition can lead to closed-loop obstruction, bowel ischemia, perforation, and peritonitis, which are associated with increased risk of death [4]. Initial imaging typically includes abdominal radiographs and computed tomography (CT) of the abdomen and pelvis, with reported sensitivities of 83% and 95%, respectively [5,6]. Multidetector CT has become the first-line imaging modality for SBO due to its ability to identify the level, severity, and cause of obstruction, detect complications such as ischemia or volvulus, and assess alternative diagnoses [6,7,8]. Intravenous contrast enhances visualization of bowel wall perfusion and mesenteric vasculature, while oral contrast may assist in certain cases with luminal delineation [9,10].

However, CT is not typically repeated once the diagnosis is established, especially in patients being managed nonoperatively. In such cases, follow-through contrast, whether performed in the radiology department via fluoroscopy or at the bedside using portable X-rays serves a distinct functional purpose: to monitor the progression of enteric contrast and help determine the likelihood of resolution without surgery. These exams are used not because they are more sensitive than CT, but because they offer a dynamic assessment of bowel function, help predict the need for operative intervention, and are better suited for serial monitoring without repeated high-dose radiation exposure [7,11].

Water-soluble contrast challenge protocols have also been shown to decrease hospital length of stay and support nonoperative decision-making, particularly when contrast reaches the colon within 24 h [8,11]. Importantly, fluoroscopic follow-through may be favored when CT is contraindicated, such as in pregnancy, contrast allergy, or renal impairment, or when cross-sectional imaging resources are limited.

Despite their utility, traditional fluoroscopic series require prolonged fluoroscopy suite occupancy and repeat staffing by radiologists, trainees, and technologists. This can create workflow bottlenecks in busy academic centers. In addition, frequent patient transport to and from the radiology department increases the risk of nosocomial infections [12,13], contributes to procedural delays [14], and may impact patient satisfaction [15]. Given that these exams are often repeated over several hours, strategies that reduce transport and decentralize imaging may improve operational efficiency and patient experience. Moreover, repeat abdominal imaging for SBO must adhere to the ALARA (As Low As Reasonably Achievable) principle, and portable serial radiography may reduce cumulative radiation dose compared to multiple CTs or fluoroscopic sessions.

To address these inefficiencies, our institution implemented a novel imaging protocol, in which the water-soluble contrast challenge is entirely performed at the bedside with follow-up portable abdominal radiographs. This approach, referred to as the Small Bowel Challenge, aims to replicate the diagnostic and therapeutic benefit of the traditional fluoroscopic series while minimizing radiology department utilization and patient movement. While the use of water-soluble contrast protocols for SBO has been previously described, prior studies have primarily evaluated protocols performed in the radiology suite [16,17,18,19,20,21]. Our study is the first to directly compare a standardized bedside contrast challenge using portable serial radiographs against the traditional fluoroscopic small bowel series, highlighting real-world differences in cost and resource utilization within the same institution [16,17,18,19,20,21].

This study is designed to compare the traditional radiology department-based small bowel series with the bedside small bowel challenge exam, focusing on differences in resource allocation, relative costs, and patient outcomes. Through statistical analysis of imaging utilization, hospital charges, and clinical endpoints, this work aims to inform diagnostic strategies that optimize resource use without compromising care quality in patients presenting with SBO.

## 2. Materials and Methods

### 2.1. Study Design

This quality improvement (QI) project was designed as a retrospective analysis to compare the efficacy and efficiency of the traditional small bowel series exam against the small bowel challenge exam. Data from patients diagnosed with small bowel obstructions (SBO) were systematically collected and analyzed to assess the outcomes of each diagnostic approach. This single-center retrospective study was conducted at a tertiary care facility. During each portable exposure, technologists used ≥2 m distance and mobile lead shields; adjacent patients/visitors were asked to step outside the doorway for the duration of the film (<60 s per film).

**Figure 1:**
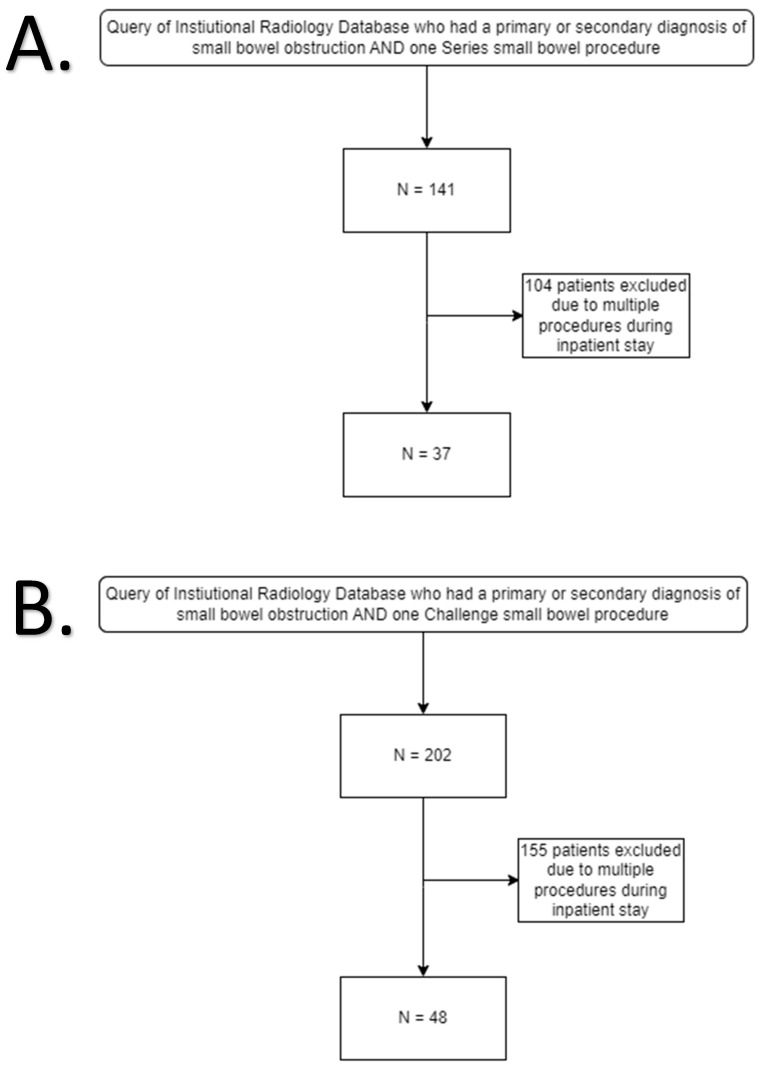
(**A**) Patient selection flow chart for Small Bowel Series group. (**B**) Patient selection flow chart for Small Bowel Challenge group.

The institutional radiology database was queried to identify inpatients diagnosed with small bowel obstruction (SBO) from 1 January 2018 to 31 December 2023. Diagnoses included “Small bowel obstruction”, “SBO (small bowel obstruction)”, “Partial small bowel obstruction”, “Small bowel obstruction due to postoperative adhesions”, “Small bowel obstruction due to adhesions”, and “Small bowel obstruction, partial”. Patients were included based on undergoing one of two specific radiological procedures during their stay: the “FL Small Bowel Series” conducted between 2018 and 2022, and the “Small Bowel Challenge” procedures administered between 2019 and 2023. Only patients who underwent a single procedure defined as small bowel challenge or series with a prior CT were retained for analysis, eliminating those with multiple interventions and resulting in 37 patients in the “Series” cohort and 47 in the “Challenge” cohort (Figure 1).

For the bedside Small Bowel Challenge protocol, patients received 200 mL of low-osmolar iodinated oral contrast (typically ioversol [Optiray 350]). This dose was not weight-based and was standardized via a pre-specified inpatient order set. High-osmolar contrast was occasionally substituted when low-osmolar contrast was unavailable. After administration of contrast, serial single-view portable abdominal radiographs were obtained at 4–6 h intervals, with a minimum of three radiographs per protocol. Additional films were performed at the discretion of the treating team if clinical concern persisted. This standardized bedside approach was designed to replicate the functional purpose of the traditional fluoroscopic small bowel series, which involves continuous radiologist supervision and a complete series of abdominal images in the fluoroscopy suite. The hospital’s internal cost-accounting system allocates fixed and variable costs using service-specific operational metrics (supply utilization, CPT codes). Clinical management after either exam, including operative versus nonoperative treatment, followed institutional guidelines and was not dictated by the choice of imaging protocol.

Cost data were obtained using the hospital’s standardized costing system, which distributes general ledger expenditures to the patient encounter and service-item level using department-specific metrics, including labor hours and revenue. This method allows for consistent estimation of both fixed and variable costs associated with radiological procedures. Fixed costs represent the baseline institutional resources required to maintain imaging capability (e.g., equipment, space, and overhead), whereas variable costs reflect resources that scale with procedure volume, such as technologist and nursing labor, medical supplies, and patient-specific pharmaceuticals.

Costs were categorized into core and non-core components. Core cost was defined as the minimum charges necessary to perform the respective diagnostic exam: for the small bowel challenge, this included three single-view abdominal radiographs with associated oral contrast; for the traditional small bowel series, this included the abdominal radiographic series with associated oral contrast. Non-core cost included all other radiology-related charges accrued during the admission, such as additional abdominal radiographs beyond the protocolized series or challenge, as well as any additional imaging or procedural materials. The primary analysis focused on total cost, defined as the sum of core, non-core, and procedure-related pharmaceutical charges, limited to the initial radiological evaluation for the index hospitalization. A secondary analysis separately assessed the frequency and costs of additional abdominal radiographs and CT examinations obtained outside of the initial diagnostic test.

All patients were required to have a preceding CT abdomen/pelvis to confirm the diagnosis of small bowel obstruction, consistent with current practice guidelines due to its higher sensitivity and overall diagnostic accuracy compared to XR [22,23]. As such, CT was used as the standard initial diagnostic modality, with either the small bowel challenge or fluoroscopic series serving as the subsequent confirmatory and potentially therapeutic exam.

Missing data were addressed through retrospective chart review; when relevant cost or imaging data were incomplete, cases were excluded from the final analysis. This approach ensured consistency in the cost calculations and prevented bias being introduced by incomplete records.

### 2.2. Statistical Analysis

The study utilized the Shapiro–Wilk test for normality, unpaired *t*-tests for parametric comparisons, and the Mann–Whitney U test for comparing non-parametric data between the two groups, with significance set at *p* values < 0.05. The analysis centered on hospital LOS, fixed, variable, and core costs, and operational metrics from the radiology department. The sample size was pulled from the data pool. As a means to better differentiate the true cost, a comparison between the core costs of the challenge versus series group was made. Descriptive statistics were reviewed to summarize patient cohorts and outcome measures.

### 2.3. Ethical Considerations

Ethical approval was secured from the Institutional Review Board (IRB) at Washington University School of Medicine. All patient data were anonymized and handled in strict accordance with HIPAA regulations to maintain confidentiality and privacy.

## 3. Results

### 3.1. Demographics

In our study, we analyzed 85 patients, with 48 in the challenge group and 37 in the series group. There was a non-significantly greater percentage of females in the challenge group (60.4%, 29/48) compared to the series group (45.9%, 17/37), while males constituted 39.6% (19/48) in the challenge group and 54.1% (20/37) in the series group. The gender distribution was not statistically significant between the two groups (*p* = 0.268). Black or African American individuals were the most represented race in both the challenge (35.4%, 17/48) and series groups (40.5%, 15/37), followed by White individuals (60.4%, 29/48 in the challenge group; 56.8%, 21/37 in the series group). Asian individuals comprised 0% (0/48) in the challenge group and 2.7% (1/37) in the series group. Native Hawaiian or Other Pacific Islander comprised 2.1% (1/48) in the challenge group. Unknown racial categories were reported at 2.1% (1/48) in the challenge group. None of the racial comparisons were significant (overall Fisher’s Exact *p* = 0.808). The mean age was 64.7 ± 13.6 years for the challenge group and 63.5 ± 17.3 years for the series group, with a range from 37 to 90 years across the study. The difference in age between the two groups was not statistically significant (*p* = 0.870). Table 1 details the demographic characteristics of patients. All patients included in the bedside small bowel challenge group successfully completed the protocol without premature termination. Tolerance was uniformly adequate, with no cases of incomplete ingestion or aborted examination reported.

### 3.2. Total Cost

Our study compared the total costs associated with the traditional small bowel series to those of the small bowel challenge exam. We included 48 cases for the challenge exam and 37 for the traditional series.

**Figure 2:**
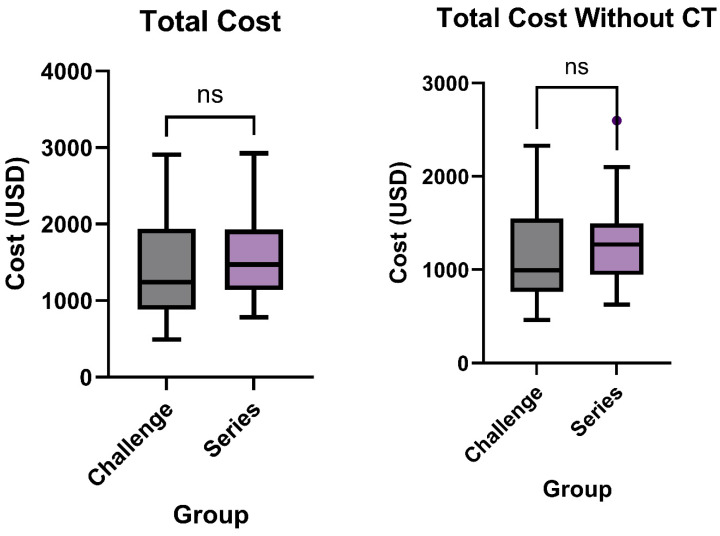
Total patient cost with and without CTs included between traditional small bowel series exam (*n* = 37) and novel small bowel challenge (*n* = 48) exam.

The median total cost for patients undergoing the small bowel challenge exam was USD 1243, with costs ranging from USD 491 to USD 2909. The mean cost was USD 1386 ± USD 614.9. For the traditional small bowel series, the median cost was higher at USD 1472, with a range from USD 783 to USD 2925, and a mean cost of USD 1530 ± USD 473.5 (*p* = 0.1229, Figure 2). While lower in the challenge group, this difference did not meet statistical significance.

Total cost was also studied after excluding CT scans from billing for each patient. The median cost for the challenge exam was USD 993.5, with a range from USD 461 to USD 2328. The mean cost in this group was USD 1138 ± USD 501.5. For the traditional series, the median cost was higher at USD 1270, with a range from USD 629 to USD 2598, and a mean cost of USD 1307 ± USD 425.8 (*p* = 0.0500, Figure 2).

### 3.3. Variable Cost

**Figure 3:**
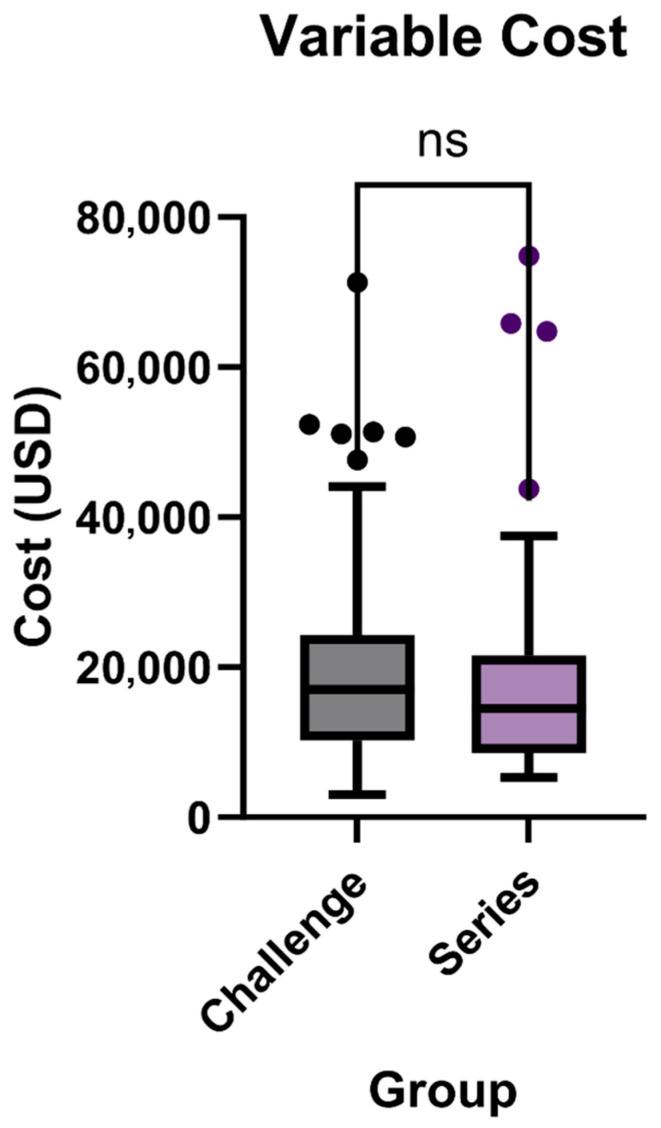
Variable costs for patients between traditional small bowel series exam (*n* = 37) and novel small bowel challenge (*n* = 48) exam (*p* = 0.3846).

Variable cost refers to total hospitalization-related charges for the index admission and not isolated imaging costs. For the challenge exam, the median variable cost was USD 17,063, with a range from USD 3044 to USD 71,332. The series had a lower median variable cost of USD 14,492, with costs ranging from USD 5277 to USD 74,824 (*p* = 0.3846, Figure 3). The mean variable cost for the challenge exam was USD 21,057 ± USD 15,404, while for the series, it was USD 19,770 ± USD 17,339.

#### Length of Stay

**Figure 4:**
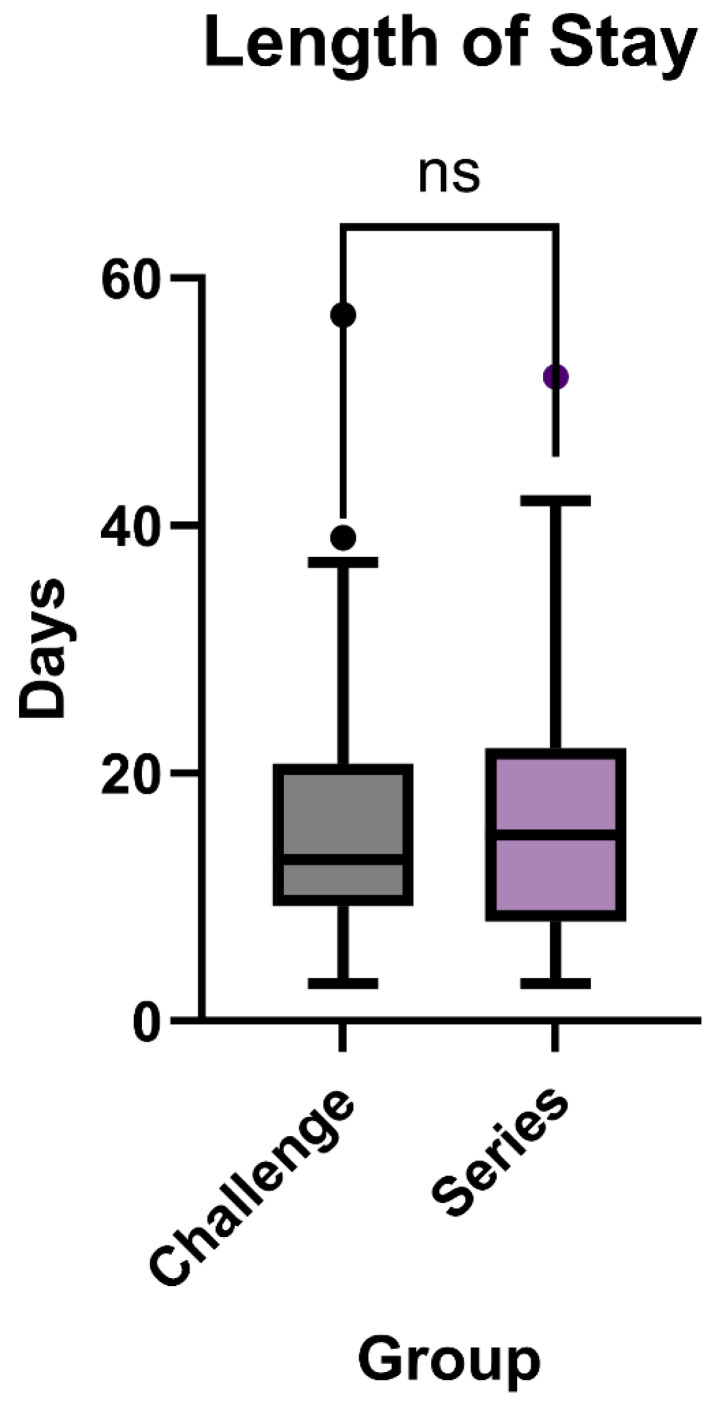
Length of stay for patients with small bowel obstruction as any diagnosis between traditional small bowel series (*n* = 37) exam and novel small bowel challenge (*n* = 48) exam (*p* = 0.8065).

In evaluating the length of stay (LOS) for patients with a primary diagnosis of small bowel obstruction, our study compared outcomes between those undergoing the small bowel challenge exam and those receiving the traditional series. The median LOS for the challenge exam group was 13 days, with a range from 3 to 57 days. The series group had a slightly higher median LOS of 15 days, with their range also spanning from 3 to 52 days (*p* = 0.8065, Figure 4).

The mean LOS was 16.48 ± 10.74 days for the challenge exam group and 17.03 ± 11.13 days for the series group.

### 3.4. Core Charges and Non-Core Charges

**Figure 5:**
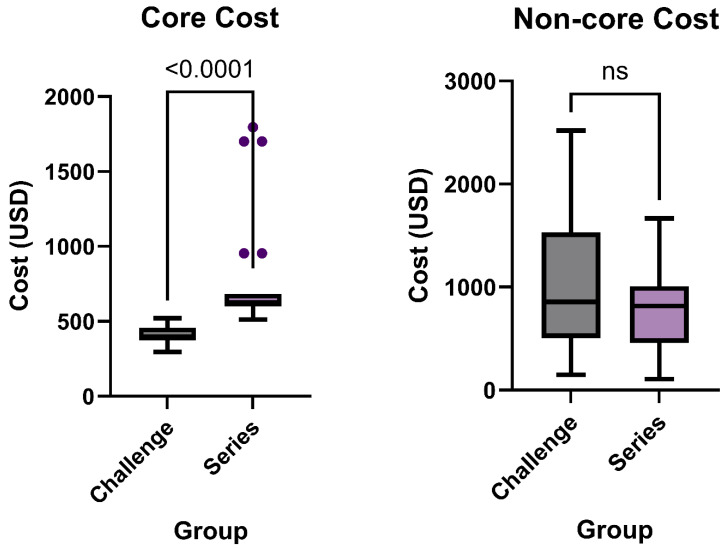
Core cost (contrast and serial imaging charges, *p* < 0.0001) and non-core cost (all other charges, *p* = 0.062) between traditional bowel series (*n* = 37) and novel small bowel exam challenge (*n* = 48).

Core cost was defined as the minimum charges required to perform the exam (contrast and protocolized abdominal radiographs), while non-core cost encompassed all other imaging-related expenses. The median for the challenge core cost which included the contrast and standard number of abdominal radiographs was USD 389.6 with a range of USD 296 to USD 521. The series group had a higher median core cost at USD 615 and range of USD 513 to USD 1796 (*p* < 0.0001, Figure 5). The mean for the core cost of the challenge group was USD 404 ± USD 58 while the mean for the series group was higher at USD 735 ± USD 309.

When comparing non-core cost, which included all additional expenses such as additional imaging and associated supplies, the median of the challenge group was USD 855.9 with a range of USD 195 to USD 2519. The median of the series group was slightly lower at USD 817 with a range of USD 107 to USD 1665 (*p* = 0.062, Figure 5). The mean for the non-core cost of the challenge group was USD 982 ± USD 593, and USD 795 ± USD 400 for the series group.

### 3.5. Additional Imaging

**Figure 6:**
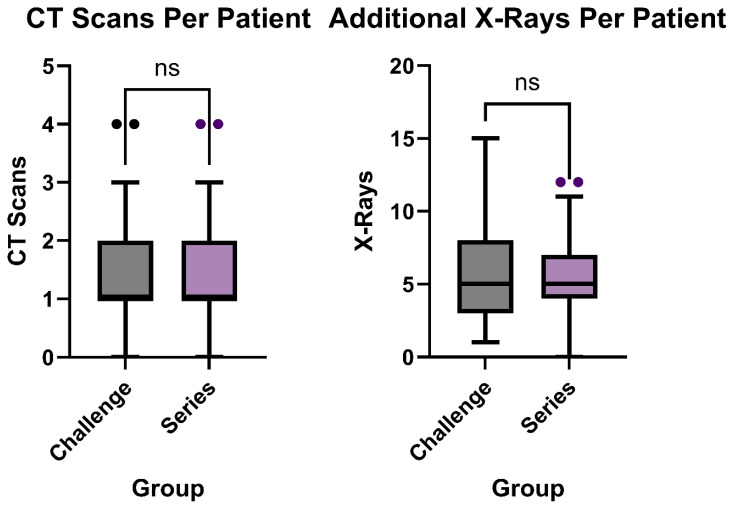
Number of CT scans (*p* = 0.96) and additional XR exams (*p* = 0.97) per patient, between traditional bowel series (*n* = 37) and novel small bowel challenge exam (*n* = 48).

The number of abdomen and pelvic CTs and additional XR exams outside of what was needed to complete the challenge during hospitalization were analyzed to compare additional radiation exposure and cost. The median CT scans per patient in the challenge group was one scan with a range of zero to four scans, which was the same for the series group (*p* = 0.96, Figure 6). The mean number of CT scans per patient in the challenge group was 1.3 ± 0.9 scans and 1.3 ± 1 scan for the series group.

For additional XR Exams per patient, the challenge group and series group both had a median 5 XR Exams, while the series group had a range of 0 to 12 XR exams and the challenge group had a range of 1 to 15 XR exams (*p* = 0.97, Figure 6). The mean number of additional XR exams per patient in the series group was 5.3 ± 3 exams, compared to 5.4 ± 3.5 exams in the challenge group.

## 4. Discussion

This retrospective quality improvement study examined the clinical and economic implications of implementing the small bowel challenge exam as an alternative to the traditional small bowel series. Although both approaches resulted in similar total costs and hospital length of stay, the challenge exam was associated with a substantial reduction in core diagnostic costs. These findings support the feasibility of adopting the challenge protocol in appropriate inpatient settings, particularly where cost containment and workflow efficiency are priorities. While our study did not directly measure radiation dose, the similar number of CT and X-ray exams between groups suggests comparable exposure. Future studies should quantify radiation dose to further evaluate potential ALARA benefits of bedside protocols. Our analysis centered on resource utilization and LOS and did not evaluate patient-centered outcomes (pain, analgesic use, satisfaction). As such, conclusions regarding symptom control or patient experience cannot be drawn from these data.

Although radiation dose was not directly measured in this study, published estimates suggest that a typical small bowel series imparts approximately 2 mSv, while a series of portable abdominal radiographs delivers around 0.7–1.0 mSv [24]. By contrast, an abdominal CT typically imparts 5–8 mSv [24]. Given that both groups underwent similar numbers of CT and X-ray examinations, cumulative exposures are expected to be comparable. Future prospective studies should include radiation dosimetry to better quantify potential ALARA benefits of bedside protocols.

The nature of the challenge exam reduces transport requirements within the hospital and GI/GU suite occupancy by allowing for portable XRs within patient rooms. This reduction could alleviate overall hospital congestion, freeing up resources for other patients. Additionally, if implemented into clinical practice and optimized, there would be no compromise in patient outcome based on the length-of-stay data collected. These findings are consistent with other studies performed at another urban academic hospital regarding length of stay but additionally found an over 40% increase in wait time to receive ordered procedures when performed in the radiology department compared to at the bedside [14].

Our findings align with reports that water-soluble contrast pathways can streamline care and lower resource use [16,17,18,19,20]. Unlike studies demonstrating shorter LOS after protocol adoption [16,17,18,19], we observed no significant LOS difference between bedside and fluoroscopic approaches. Possible explanations include our inclusion of all inpatients, mandatory preceding CT in both groups, and real-world protocol heterogeneity. Notably, our core cost reduction with bedside implementation extends prior work by quantifying radiology-specific cost components within a single institution.

Patient satisfaction, though not directly measured in this study, is a major component of building the physician–patient relationship in the health care system, and is a major consideration, especially when discussing wait times. A recent study looking at the preference for procedure location found that patient surveys consistently stated that the bedside was chosen due to increased comfort compared to the radiology department [15]. Decreasing patient transit time and increasing bedside procedure time can be conducive to enhancing patient comfort and satisfaction, as well as potentially decreasing risk of nosocomial infections [12,13]. This, coupled with no increase in LOS, supports the challenge exam as a resource-efficient alternative to the fluoroscopic series, and clinical superiority was not established in this study.

Although the bedside small bowel challenge is generally well tolerated, potential risks such as aspiration and perforation should be considered. While these complications were not observed in our cohort, they are described in the literature and must be carefully weighed when implementing bedside protocols. We recommend that institutions adopting this approach develop standardized monitoring guidelines to ensure patient safety.

These conclusions align with an evolving body of literature supporting standardized water-soluble contrast challenge protocols for SBO. The American College of Radiology recommends such protocols as part of streamlined diagnostic pathways, noting that they can often be performed at the bedside or in a single imaging location, thereby expediting disposition while reducing the need for serial transport [25]. Multi-institutional studies have demonstrated that these protocols are safe, effective, and reduce unnecessary interventions and readmissions [18,20,26,27].

Survey data suggest that the water-soluble contrast challenge is widely adopted among academic centers for nonoperative SBO management, with most studies performed in a single location [28]. Such practices allow for early decision-making on operative versus conservative management and may reduce delays in care [29]. Our results reinforce the viability of incorporating this method into inpatient workflows without compromising clinical outcomes.

Existing data also supports the clinical and financial advantages of these protocols. Prior studies have shown that implementation of such protocols reduces average hospital length of stay, with nonoperative LOS as low as 2 to 5 days and significant cost savings in both adult and pediatric populations [16,17,18]. For instance, one study noted a decrease in mean LOS from 8.3 to 4.8 days following protocol adoption [16], while another reported a 49% reduction in total hospital costs using emergency department-based contrast challenge protocols, with a median LOS under 24 h for select patients [19]. Importantly, these benefits were achieved without increases in complication or readmission rates [19,20]. Recent work has also identified laboratory markers such as the direct bilirubin-to-lymphocyte ratio as promising tools for predicting surgical need in adhesive SBO [21]. Our study supports these findings, demonstrating that the bedside contrast challenge is a viable, lower-cost alternative to the fluoroscopic series without compromising clinical outcomes.

This study is not without limitations. One limitation of this study is the small sample size of the series and challenge groups. Furthermore, this study was conducted at a single academic center, which can limit generalizability. Although data was primarily collected concerning cost analyses for this study, we were unable to collect data regarding patient perceptions and specific quantification of transit times and transport costs due to difficulty obtaining the data. Additionally, data on complications and readmissions were not consistently available in the retrospective dataset. While complications were likely captured indirectly through their effect on measured variables such as cost and length of stay, readmissions represent separate encounters and therefore could not be assessed in this analysis.

Although we attempted to use strict inclusion and exclusion criteria, the nature of a retrospective analysis may affect the availability and overall accuracy of medical records. This study does offer the real-world implementation of changing a radiology practice pattern when imaging SBO patients with oral contrast agents. In addition to the small sample size and single-center setting, our analysis did not quantify radiation dose, capture complication/readmission rates, or measure patient satisfaction. Accordingly, the present study should not be interpreted as demonstrating differences in pain control or other patient-centered outcomes.

Future directions should focus on a multi-center approach to enhance generalizability and increase sample size. A more comprehensive analysis should include both qualitative data on patient perceptions and standardized questionnaires to assess satisfaction, alongside quantitative data such as transit times and cumulative radiation exposure to evaluate operational efficiency. Finally, scalability of the protocol to non-academic settings should be examined, including portable radiography capacity, staff training, and contrast availability in community hospitals.

## 5. Conclusions

Overall, we suggest that the small bowel challenge exam offers a lower-utilization method to streamline resource management in a hospital setting for small bowel obstruction patients rather than performing a small bowel series. The small bowel challenge exam demonstrated a significantly lower core cost compared to the small bowel series exam and did not significantly affect the overall length of stay for patients. While our study demonstrates significant reductions in diagnostic costs with the bedside challenge protocol, further research is needed to establish safety outcomes before definitive cost-effectiveness can be claimed. Our results in this study should therefore be interpreted as evidence of cost reduction rather than full cost-effectiveness. Future multi-center analysis via quantitative assessment of transport times and qualitative analysis of patient perceptions can be beneficial to further validate the small bowel challenge as a valid, less-burdensome alternative to the small bowel series exam both from a radiology and an overall hospital perspective.

## Figures and Tables

**Table 1:** General Demographic Characteristics for patients in bowel challenge vs. series groups.

Demographics	Challenge Group	Series Group	*p* Value
Gender			0.268
Female	60.4% (29)	45.9% (17)	
Male	39.6% (19)	54.1% (20)	
Race			0.808
Asian	0% (0)	2.7% (1)	
Black or African American	35.4% (17)	40.5% (15)	
Native Hawaiian or Other Pacific Islander	2.1% (1)	0% (0)	
Unknown	2.1% (1)	0% (0)	
White	60.4% (29)	56.8% (21)	
Age (years)	64.7 ± 13.6	63.5 ± 17.3	0.870

## Data Availability

Dataset available on request from the authors. Dataset restricted due to Health Insurance Portability and Accountability Act.
